# Pediatric Urology highlighted

**DOI:** 10.1590/S1677-5538.IBJU.2020.03.01

**Published:** 2020-02-20

**Authors:** Luciano A. Favorito

**Affiliations:** 1 Universidade do Estado de Rio de Janeiro Unidade de Pesquisa Urogenital Rio de JaneiroRJ Brasil Unidade de Pesquisa Urogenital - Universidade do Estado de Rio de Janeiro - Uerj, Rio de Janeiro, RJ, Brasil; 2 Hospital Federal da Lagoa Serviço de Urologia Rio de JaneiroRJ Brasil Serviço de Urologia, Hospital Federal da Lagoa, Rio de Janeiro, RJ, Brasil

The May-June number of *Int Braz J Urol*, the 3^rd^ under my supervision, presents original contributions with a lot of interesting papers in different fields: Prostate Cancer, Renal Cell Carcinoma, Penile trauma, Bladder Cancer, Neurogenic Bladder, Robotics, Laparoscopy, Male Health, Ureteroscopy, Hypospadia, Urinary diversion and Testicular Cancer. The papers came from many different countries such as Brazil, USA, Turkey, China, Montenegro, Spain, India and Italy, and as usual the editor's comment highlights some of them. Pediatric urology is the highlight of this number. In the present issue we present two important reviews: in page 314 Dr. Kirsch and Arlen from Atlanta - USA (1) review in a nice narrative if the open ureteral reimplantation is still the gold standard treatment in surgical management of vesicoureteral reflux and the authors concluded that open, laparoscopic/robot- assisted and endoscopic approaches are all successful in correcting re ux and have been shown to reduce the incidence of febrile urinary tract infections and Dr. Fuchs and Dajusta and from Columbus – USA (2) present in page 322 an important review about robotics in Pediatric urology and concluded that several procedures in Robotic has also shown feasibility and comparable success when compared to open surgery in procedures that were previously deemed too complex to be done by standard laparoscopy.

Dr. Azambuja and collegues from Brazil performed on page 353 (3) an important retrospective study about the treatment of testicular cancer with platinum combinations in 50 patients. The authors concluded that the expression of excision repair cross-complementation (ERCC1) and nuclear factor kappa-B give a worse prognosis for relapse, and only ERCC1 had an influence on the overall survival of Testicular germ cells tumor patients treated with platinum-based chemotherapy. These may represent markers that predict poor clinical outcome and response to cisplatin.

Dr. Sharma and Collegues from India performed on page 363 (4) a very important study about the use of androgen deprivation therapy (ADT) in carcinoma prostate (CaP) and if the ADT has deleterious effect on bone mineral density (BMD) leading to increase incidence of osteoporosis and skeletal-related events in 96 patients and concluded that the Bone-directed therapy (Zoledronic acid) leads to both subjective and objective improvement in bone health of prostate cancer patients on ADT.

Dr. Taha and collegues from Brazil studied patients over 60 years of age, to obtain data on its sexual and urinary health with 3425 questionnaires and found a large number of sexual and urinary disorders and recommended the improvement in health conditions, promoting a better quality of life in the elderly (5).

Dr. Freitas and collegues from Brazil performed on page 383 (6) another study about Androgen deprivation therapy (ADT) in advanced prostate cancer and compared efficacy of three schedules of leuprolide acetate in lowering PSA in a real world population in 932 patients during 11 years and concluded that PSA levels can be effectively be reduced in most patients treated with monthly, quarterly, or semiannual injections of long-acting leuprolide acetate.

Dr. Fedrigon and Collegues from USA performed on page 390 (7) a in vitro study about two automated irrigation systems for use during ureteroscopy and concluded that each system provided steady irrigation at safe pressures within their expected operating parameters with small differences in performance that should not limit their ability to provide steady irrigation at safe pressures.

Dr. Barros and Collegues from Rio de Janeiro- Brazil demonstrated in page 409 (8) in a very nice paper the experience over the past 20 years in the diagnosis and surgical treatment of penile fracture (PF) in 255 patients and concluded that penile fracture has typical clinical presentation and no need for additional tests in most cases. The ‘doggy style’ and ‘man-on-top’ was the most common positions and generally associated with more severe lesions. Concomitant urethral injury should be considered in cases of high-energy trauma. Surgical reconstruction produces satisfactory results, however, it can lead to complications, such as erectile dysfunction and penile curvature.

Dr. Kalil and Dr. D'ancona in page 419 (9) in a very important paper evaluated the lower urinary tract symptoms, classified by the International Prostate Symptom Score (IPSS), urodynamic results, Watts Factor (WF), Bladder Contractility Index (BCI), and post void residual (PVR), in order to differentiate Detru- sor Underactivity (DU) from Bladder Outlet Obstruction (BOO) in 44 patients and concluded that isolated symptoms, classified by IPSS and PVR, could not differentiate patients with DU from those with BOO, but it was possible using urodynamic data.

Finally, Dr. Kavaric and Collegues from Montenegro performed on page 446 (10) the study that is on the cover in this number. The authors compared perioperative outcomes, complications and anastomotic stricture rate in a contemporary series of patients who underwent open radical cystectomy (RC) with modified Wallace anastomotic technique versus traditional ileal conduit in 140 randomized patients and concluded that the use of the modified Wallace technique leaves to significantly lower anastomotic stricture and anastomotic leakage rates, which are major issues in minimizing both short and long-term postoperative complications.

We hope that readers will enjoy the present number of the *International Brazilian Journal of Urology*.
